# Ferric reductase related proteins Frp1 and Frp2 mediate reductive heme uptake in the opportunistic pathogen *Candida albicans*^☆^

**DOI:** 10.1016/j.jinorgbio.2026.113246

**Published:** 2026-02-01

**Authors:** Yibo Fu, Yungeun Lee, Daniel Kornitzer, Amit R. Reddi

**Affiliations:** a School of Chemistry and Biochemistry, Georgia Institute of Technology, Atlanta, GA 30332, United States of America; b School of Biological Sciences, Georgia Institute of Technology, Atlanta, GA 30332, United States of America; c Parker Petit Institute for Bioengineering and Biosciences, Georgia Institute of Technology, Atlanta, GA 30332, United States of America; d Department of Molecular Microbiology, B. Rappaport Faculty of Medicine, Technion - Israel Institute of Technology, Haifa, Israel

**Keywords:** Heme, Iron, *Candida albicans*, Reductive heme uptake, *Saccharomyces cerevisiae*

## Abstract

Heme is an essential cofactor and nutritional source of iron for many pathogenic microbes, including the opportunistic fungal pathogen *Candida albicans*. *C. albicans* utilizes common fungal extracellular membrane (CFEM) domain containing hemophores to scavenge heme from the host and deliver it across the cell wall to the cell membrane, where is it subsequently internalized. Previous studies indicated that ferric reductase related proteins Frp1 and Frp2 were required for hemin transport and utilization. However, the molecular mechanism of Frp1 and Frp2 dependent heme uptake remains to be fully elucidated. Motivated by the similarity of Frp1 and Frp2 to ferric iron reductases, we sought to determine if Frp1/2 exhibited heme reductase activity and if heme‑iron oxidation state influenced its uptake in *C. albicans*. By utilizing ratiometric heme sensors, redox inactive fluorescent ferric and ferrous heme analogs, and a modified pyridine hemochrome method, we determined that *C. albicans* has a preference for transporting ferrous heme over ferric heme and exhibits cell surface heme reductase activity that is in part dependent on Frp1 and Frp2. Our results are the first to directly demonstrate *C. albicans* has the capacity to reduce extracellular heme‑iron and that its oxidation state may be important for heme uptake. We further found that another yeast species, *Saccharomyces cerevisiae*, which poorly utilizes heme iron, also exhibits heme reductase activity, pointing to the broader presence of extracellular heme reduction in the microbial world.

## 1. Introduction

*Candida albicans* (*C. albicans*) is a commensal fungal pathogen that inhabits the oral cavity, skin, and gastrointestinal tracts of humans (1). As an opportunistic pathogen, *C. albicans* is otherwise harmless unless host immune status, barrier integrity and/or the microbiome are disrupted, which can lead to invasive growth and ultimately life-threatening yeast infections (2). As part of this transition from being commensal to pathogenic, *C. albicans* morphology changes from a budding state to a more invasive hyphal form, which can pierce and penetrate peripheral tissues (3–5). *C. albicans* is the most common *Candida* species responsible for invasive candidiasis (6).

A key defense strategy to limit the growth of pathogenic microbes is for the host to limit access to essential nutrients, a phenomenon known as nutritional immunity (7). Iron is one such essential trace element that is important for the virulence of *C. albicans*. Within the human host, both heme and non-heme iron can be scavenged by *C. albicans*, with heme iron being the most abundant form of iron, accounting for more than 80% of total iron in humans (8,9). Non-heme iron is largely oxidized and insoluble, limiting its bioavailability. One mechanism to scavenge limiting amounts of extracellular Fe^3+^ is deployment of secreted high affinity iron chelators, i.e. siderophores, to bind the limiting amounts of soluble Fe^3+^. While *C. albicans* cannot synthesize siderophores, it expresses receptors to scavenge siderophores produced by other microbes. Another mechanism to scavenge iron is to utilize ferric iron reductases (FRE) at the cell surface to reduce insoluble ferric iron to more soluble ferrous iron to facilitate its transport across the membrane. Indeed, *C. albicans* encodes multiple FRE like protein genes including Fre1/Cfl1 and Fre10 (10,11).

Heme iron is more bioavailable than non-heme iron. This is in part because the heme iron center is bound tightly and solubilized by the protoporphyrin IX macrocycle and heme‑iron accounts for more than 80% of total iron in mammals (12,13). Thus, heme scavenging represents a major source of iron for many pathogenic microbes, including *C. albicans* (13). Heme acquisition by *C. albicans* involves the use of common in fungal extracellular membrane (CFEM) domain containing hemophores that contain a conserved aspartate residue that selectively coordinates ferric heme (14–16). CFEM hemophores include the soluble hemophore Csa2, and the cell wall and plasma membrane glycosylphosphatidylinositol (GPI)-anchored hemophores Rbt5 and Pga7, respectively (17–20). Free heme or heme acquired from hemoglobin is exchanged between the extracellular CFEM hemophores Csa2, Rbt5 and Pga7, and ultimately internalized via a mechanism that depends on the transmembrane proteins Frp1 and Frp2 (17).

On the basis of their sequence similarity with ferric reductase (FRE) proteins, including having nicotinamide adenine dinucleotide (NADH) or nicotinamide adenine dinucleotide phosphate (NADPH), flavin adenine dinucleotide (FAD), and heme binding sites (17), Frp1/2 may serve to reduce extracellular hemin (Fe^3+^-heme). In such a scenario, and analogous to non-heme iron reduction by FRE proteins, Frp1/2 catalyzes the reduction of extracellular Fe^3+^-heme using intracellular NADPH by way of redox cofactors FAD and heme. As such, there may be a reductive pathway for *C. albicans* heme assimilation. Herein, by utilizing genetically encoded ratiometric heme sensors, a newly developed heme reductase assay, and redox-inactive fluorescent heme analogs of Fe^3+^- and Fe^2+^-heme, we found that *C. albicans* has a preference to take up ferrous over ferric heme and that Frp1/2, at least in part, mediates the reduction of extracellular hemin. Altogether, *C. albicans* exhibits a robust heme reductase activity, which may be important for reductive heme uptake and assimilation.

## 2. Results

### 2.1. C. albicans prefers to uptake ferrous heme over ferric heme

**Fig. 1.**
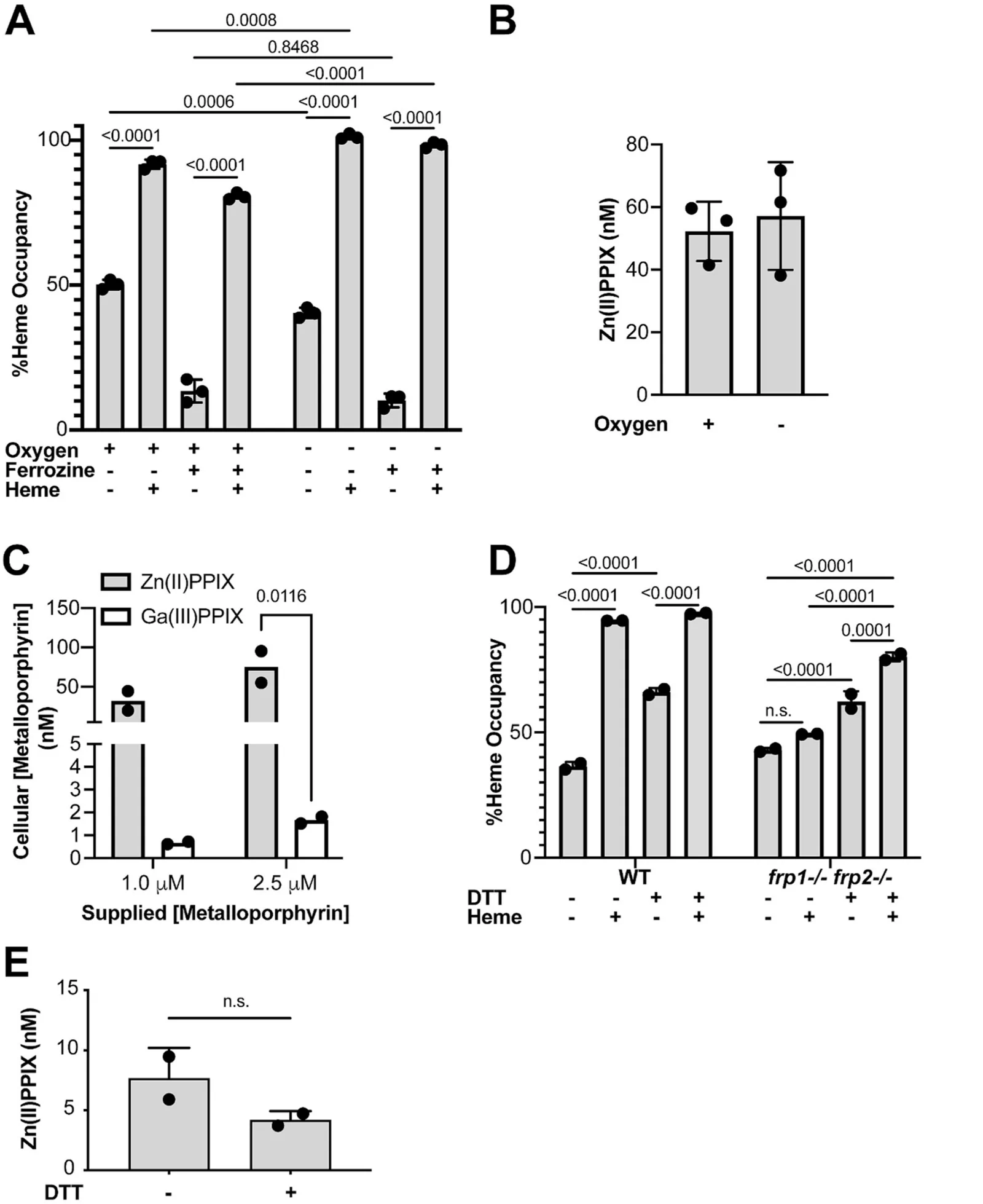
Reductive heme uptake in *C. albicans*. (A) The effects of ferrozine (1 mM) and oxygen on uptake of hemin (0.5 μM) over 90 min in wild type *C. albicans* expressing the HS1-M7A heme sensor. (B) Effect of oxygen on uptake of 1 μM zinc(II)protoporphyrin IX (Zn(II)PPIX) uptake over 2 h. (C) Relative accumulation of the indicated concentration of Zn(II)PPIX versus Ga(III)PPIX in *C. albicans*. (D) The effects of DTT (1 mM) on uptake of hemin (0.5 μM) in wild type and *frp1*^−/−^ *frp2*^−/−^ *C. albicans* expressing the HS1-M7A heme sensor. (E) The effects of DTT (1 mM) on uptake of 1 μM Zn(II)PPIX in *C. albicans*. In panel A, the data reflect the mean ± standard deviation (SD) of triplicate measurements and the statistical significance assessed by a two-way ANOVA with the Tukey's post-hoc. In panel B, the data reflect the mean ± standard deviation (SD) of triplicate measurements and the statistical significance assessed by a two-tailed *t*-test. In panel C, the data reflect the mean ± standard deviation (SD) of duplicate measurements and the statistical significance assessed by a two-way ANOVA with the Tukey's post-hoc. In panel D, the data reflect the mean ± standard deviation (SD) of triplicate measurements and the statistical significance assessed by a two-way ANOVA with the Tukey's post-hoc. In panel E, the data reflect the mean ± standard deviation (SD) of duplicate measurements and the statistical significance assessed by a two-tailed *t*-test. All experiments were conducted in at least two independent experimental trials, with the data plotted being representative. In all panels, n.s. denotes “not significant”.

To probe the oxygen dependence of heme uptake in *C. albicans*, yeast expressing genetically encoded heme sensor HS1-M7A (21) were cultured with or without oxygen, 1 mM ferrozine, an iron chelator, and/ or 0.5 μM hemin chloride at 30 °C in YPD media for 90 min (16,17). As shown in Fig. 1A, under oxygen replete conditions, sensor heme occupancy increased from ~50% to ~90% upon hemin supplementation, decreased from ~50% to ~13% upon ferrozine supplementation, and increased from ~13% to ~80% when hemin was added back to ferrozine supplemented cells. These results were exactly as expected since *C. albicans* has a robust heme uptake system and iron is required for heme synthesis. In the absence of oxygen, sensor heme occupancy exhibited a modest, albeit statistically significant decrease from 50% to 40%, which was expected due to the oxygen-dependence of heme synthesis (Fig. 1A). However, in the absence of oxygen, sensor detectable heme uptake was significantly enhanced, by as much as 20%. Sensor heme occupancy increased from ~40% to ~100% upon hemin supplementation in the absence of oxygen, while in the presence of oxygen it increased from ~50% to ~90%, accounting for a net ~ +20% increase in sensor heme occupancy under anoxic conditions (Fig. 1A). Likewise, under iron deficiency (+ ferrozine), sensor heme occupancy increased from ~10% to ~98% upon hemin supplementation in the absence of oxygen, while in the presence of oxygen it increased from ~13% to ~80%, accounting for a net ~ +20% increase in sensor heme occupancy due to anoxia (Fig. 1A).

The apparent increase in heme uptake in the absence of oxygen suggested the presence of an oxygen dependent mechanism for heme transport. One possibility is that heme uptake factors may be induced under hypoxia or anoxia. Indeed, certain *C. albicans* hemophores, including Rbt5 and Pga7, were previously found to be induced under oxygen limited growth (22). To determine if this was the case in our experiments, we assessed if there was an oxygen-dependence to the uptake of the redox inactive fluorescent heme analog zinc(II)protoporphyrin IX (Zn(II)PPIX). Zn(II)PPIX and other fluorescent metalloporphyrins are commonly used to probe heme uptake in various cells and organisms (23–29) and we reasoned that if there was an inducible heme uptake system, we should also observe elevated accumulation of Zn(II)PPIX under hypoxic conditions. However, we observed a similar degree of Zn(II)PPIX accumulation under normoxic and anoxic growth that was statistically indistinguishable (Fig. 1B). Together, these data suggest that a general oxygen-dependent inducible heme uptake system is not responsible for the elevated heme uptake observed under anoxia (Fig. 1A) and that the redox active iron center may be critical for enabling oxygen dependent heme uptake (Fig. 1B).

One model that explains the observation that heme uptake is oxygen dependent and enhanced under oxygen limiting conditions is that ferrous Fe^2+^-heme is a better substrate for transport than ferric Fe^3+^- heme. To test this idea, we assessed if *C. albicans* exhibited a preference to internalize Zn(II)PPIX over gallium(III)protoporphyrin IX (Ga(III) PPIX). Ga(III)PPIX and Zn(II)PPIX are redox inactive fluorescent heme analogs that potentially serve as mimics of Fe^3+^- or Fe^2+^-heme, respectively, due to similar metal oxidation numbers and effective ionic radii (30,31). For example, 6-coordinate high spin Fe(III) and Fe(II) complexes have effective ionic radii of 0.645 Å and 0.780 Å, respectively, while 6-coordinate Ga(III) and Zn(II) have effective ionic radii of 0.620 Å and 0.740 Å, respectively (30). We found that Zn(II) PPIX accumulated in *C. albicans* to a far greater extent (more than 50-fold in 2 h) than Ga(III)PPIX (Fig. 1C), suggesting that reduced ferrous Fe^2+^-heme may be a better substrate for transport and internalization than oxidized ferric Fe^3+^-heme.

One important caveat is that, despite the comparable ionic radii and charge of Fe(II)- and Fe(III)-PPIX to Zn(II)- and Ga(III)-PPIX, respectively, the fluorescent metallporphyrins are still chemically distinct from iron containing heme/hemin. To further substantiate that *C. albicans* has a preference to uptake ferrous over ferric heme, we determined if supplementation with the reducing agent dithiothreitol (DTT) (32,33) could enhance heme uptake in cells expressing the heme sensor HS1 (21). To maximize the dynamic range of the sensor and the change in signal due to heme binding, cells were cultured with 1 mM ferrozine to deplete intracellular heme to minimize heme sensor occupancy. Since DTT in the presence of oxygen can potentially generate oxygen radicals (34), which can confound interpretation of heme uptake results, experiments were performed in the absence of oxygen. As shown in Fig. 1D, heme sensor occupancy of WT *C. albicans* increased from ~35% to ~95% upon supplementation with 0.5 μM hemin. Supplementation of cells with DTT alone increased sensor heme occupancy from ~35% to ~66%, suggesting that reductive stress could mobilize intracellular heme stores. Moreover, given that HS1 binds reduced heme more tightly than oxidized heme (21), DTT-mediated reduction of intracellular heme could result in increased HS1 heme occupancy. In the presence of heme and DTT, sensor heme occupancy was ~95%, similar to heme supplementation without DTT.

Since heme uptake appeared to already be saturating in WT cells, we sought to determine if DTT exerted an effect on cells lacking *frp1*^−/−^ *frp2*^−/−^. Frp1 and Frp2 were previously shown to be required for heme uptake (17). Indeed, we observed that hemin supplementation resulted in minimal uptake of heme in *frp1*^−/−^ *frp2*^−/−^ cells compared to WT (Fig. 1D). However, DTT supplementation resulted in a more substantial accumulation of heme in *frp1*^−/−^ *frp2*^−/−^ cells. Since Frp1 and Frp2 are similar to ferric iron reductases, we surmised that DTT chemically complemented Frp1/2 to facilitate heme reduction and its subsequent uptake. To rule out that the enhanced uptake of heme was due to DTT-mediated reduction of cell membrane or cell wall proteins and general changes in membrane permeability (35), we determined if DTT affected uptake of the fluorescent heme analog Zn(II)PPIX. As shown in Fig. 1E, the addition of DTT did not enhance accumulation of Zn(II)PPIX, thereby ruling out general DTT-dependent changes in membrane permeability to increase heme uptake. Moreover, the concentration and exposures of DTT utilized did not affect cell viability (data not shown). Nevertheless, we cannot completely rule out confounding contributions associated with DTT and reductive stress. Taken together, the results herein suggest that oxygen affects heme uptake and that this may be mediated by changes in heme iron oxidation state, with Fe^2+^-heme being a better substrate for transport than Fe^3+^-heme.

### 2.2. C. albicans exhibits heme reductase activity

**Fig. 2.**
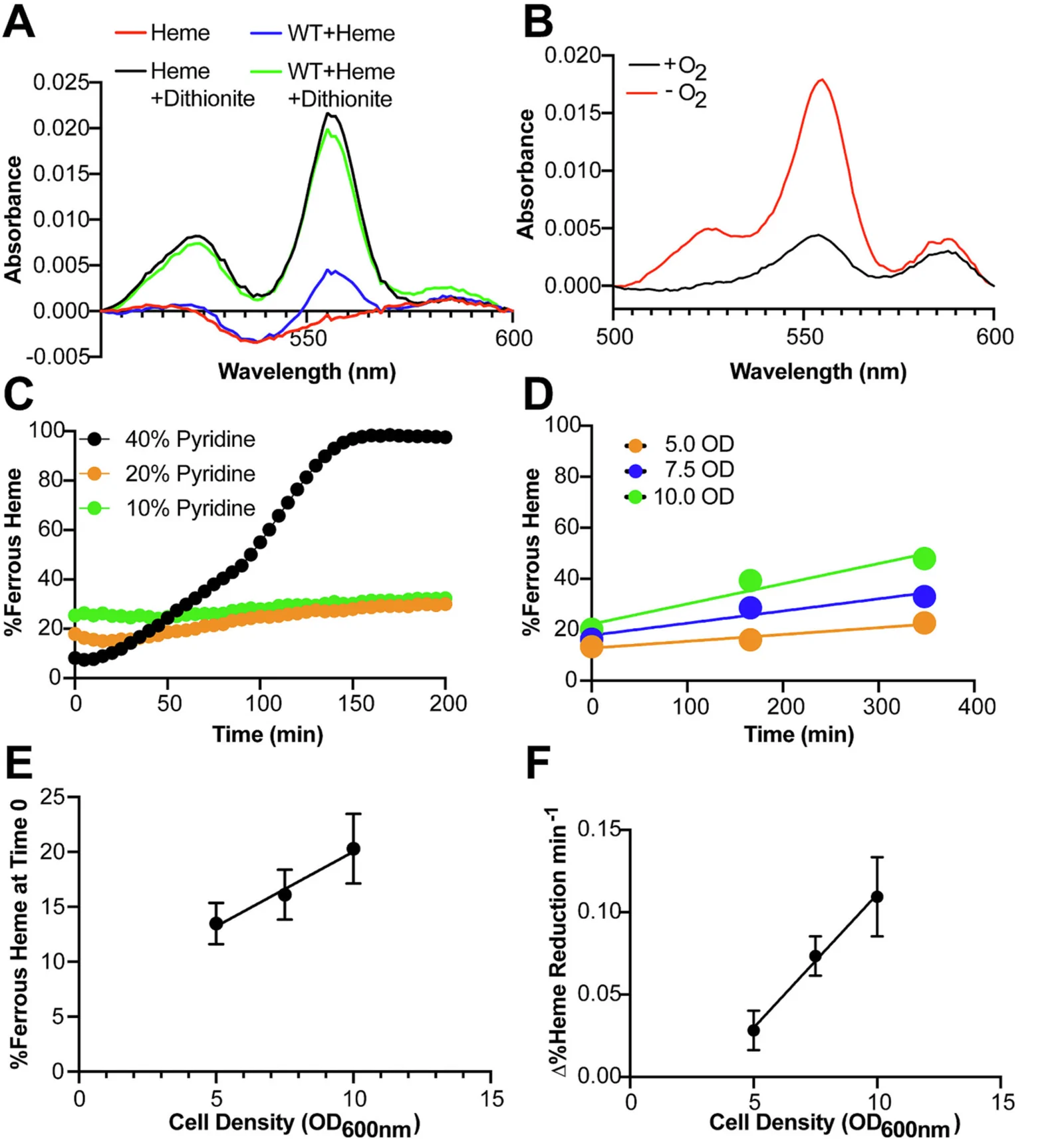
*C. albicans* exhibits a heme reductase activity. (A) WT *C. albicans* has the capacity to reduce extracellular hemin as assessed by the pyridine hemochrome method. 5 μM hemin was incubated with or without 10 mM dithionite and/or with or without 10 OD_600nm_ of WT *C. albicans* for 60 min prior to measurement of the UV/visible spectra with pyridine in PBS. (B) *C. albicans* reduced heme is re-oxidized in the presence of oxygen. UV/vis spectra of heme with pyridine in PBS after co-incubation with 20 OD_600nm_ of WT *C. albicans* for 360 min in an anaerobic chamber or after its exposure to atmospheric oxygen for less than 1 min. (C) Effect of the pyridine concentration used to extract heme from the cell pellet on pyridine hemochrome stability as assessed by the change in % heme reduction as monitored by UV/visible spectroscopy. (D) Cell number dependence on % heme reduction over time. (E) Cell number dependence of initial heme reduction at time 0 (less than 5 min of exposure to heme). (F) The change in percent heme reduction per minute as a function of cell number as determined from the slopes of the plot depicted in panel D. Panel D is representative of three independent trials. In panels E and F, the data reflect the mean ± standard deviation (SD) of triplicate measurements and is derived from the type of plot depicted in panel D, which shows a representative single trial. For panel E, linear regression analysis indicates *p* = .0094 and a goodness of fit (R^2^) of 0.64. For panel F, linear regression analysis indicates *p <* .0001 and a goodness of fit (R^2^) of 0.85.

Since *C. albicans* prefers to transport ferrous over ferric heme, we next sought to determine if yeast could reduce extracellular ferric heme. To detect extracellular heme reduction, cells were incubated with hemin chloride (Fe^3+^-heme) in an anaerobic chamber and extracellular heme reduction was measured using pyridine, which binds ferrous heme selectively over ferric heme and exhibits a distinct UV/visible spectrum when bound to ferrous heme (25,36,37). As shown in Fig. 2A, incubation of 5 μM hemin chloride (Fe^3+^-heme) in phosphate buffered saline (PBS) with 40% pyridine did not result in an observable UV/vis signature between 500 nm and 700 nm. However, when hemin chloride was co-incubated with sodium dithionite, a strong reducing agent, the distinct α and β Q-bands of reduced Fe^2+^-heme emerge between 500 nm and 600 nm, with absorbance maxima at 525 nm and 557 nm. When a 500 μL suspension of 10 OD_600nm_ of WT *C. albicans*, corresponding to 2.0 × 10^8^ cells (38), was incubated with hemin chloride for 60 min, a UV/visible spectrum consistent with formation of reduced heme emerged, even though no exogenous reductants were added. Addition of sodium dithionite to the sample containing hemin chloride and cells resulted in emergence of a fully reduced heme spectra similar to that of hemin chloride and dithionite in PBS buffer. The spectra obtained with sodium dithionite reflects total heme in the sample and the fraction or percentage of heme reduced can be determined by calculating the ratio of the absorbance intensities at 557 nm without and with sodium dithionite. Taken together, *C. albicans* has the capacity to reduce extracellular heme.

Of note, when yeast cells are incubated with hemin, most of it adheres to the cell surface. Pelleting of cells results in an undetectable amount of heme in the supernatant using the pyridine hemochrome method, even with sodium dithionite (data not shown). To extract cell surface associated heme, cells are pelleted, and the pyridine hemochrome solution (PHS) is used to resuspend the cells and extract surface associated heme. Cells are re-pelleted and the solubilized heme in the PHS is removed and the pyridine hemochrome signal is measured with or without dithionite.

As expected, cell-mediated heme reduction is reversible and dioxygen can re-oxidize heme. Co-incubation of hemin with 20 ODs of cells for 360 min in an anaerobic chamber followed by heme extraction with PHS results in the characteristic spectra of a fraction of the heme being reduced (Fig. 2B). However, when the PHS heme extract was exposed to atmospheric oxygen for ~1 min, there was an attenuation of the ferrous heme signal at 557 nm, consistent with air oxidation of heme (Fig. 2B). Thus, while cells have the capacity to actively reduce heme, a fraction of it can be re-oxidized by oxygen, which in turn can potentially affect cellular heme import (see discussion).

We next sought to optimize pyridine content in the heme reductase assay. Since pyridine can compromise cell membrane integrity (39), we sought to determine the minimum concentration required to measure apparent heme reductase activity. As shown in Fig. 2C, the pyridine hemochrome signal at 557 nm increased over time with 40% pyridine, while staying relatively constant with 20% or 10% pyridine. We attributed the instability of the pyridine hemochrome signal at high concentrations of pyridine to cell lysis and release of small molecules that could affect formation of the pyridine hemochrome. Therefore, 10% pyridine was selected as the standard pyridine concentration to work with for subsequent heme reductase measurements.

Incubation of 5–10 OD_600_/mL of cells with 5 μM hemin resulted in a dose dependent increase in heme reduction over a period of 350 min (Fig. 2D). The data show a a fast reduction step, which occurs within the ~5-min processing time for measuring cellular heme reduction, and is indicated by the y-intercept at time “0” (Fig. 2D). The initial fast reduction is proportional to cell number, with 5.0, 7.5, and 10.0 OD_600nm_ of cells resulting in 13%, 16%, and 20% heme reduction, respectively (Fig. 2E). Following this, there is a slower linear increase in heme reduction up to 350 min (Fig. 2D). Calculation of the slope gives the change in % heme reduction per unit of time (Δ%Heme Reduction min^−1^), which we refer to as “heme reductase activity”. As demonstrated in Fig. 2F, heme reductase activity is linear with cell number between 5 and 10 OD_600nm_.

### 2.3. C. albicans heme reductase activity is in-part dependent on Frp1 and Frp2 and carbon source

**Fig. 3.**
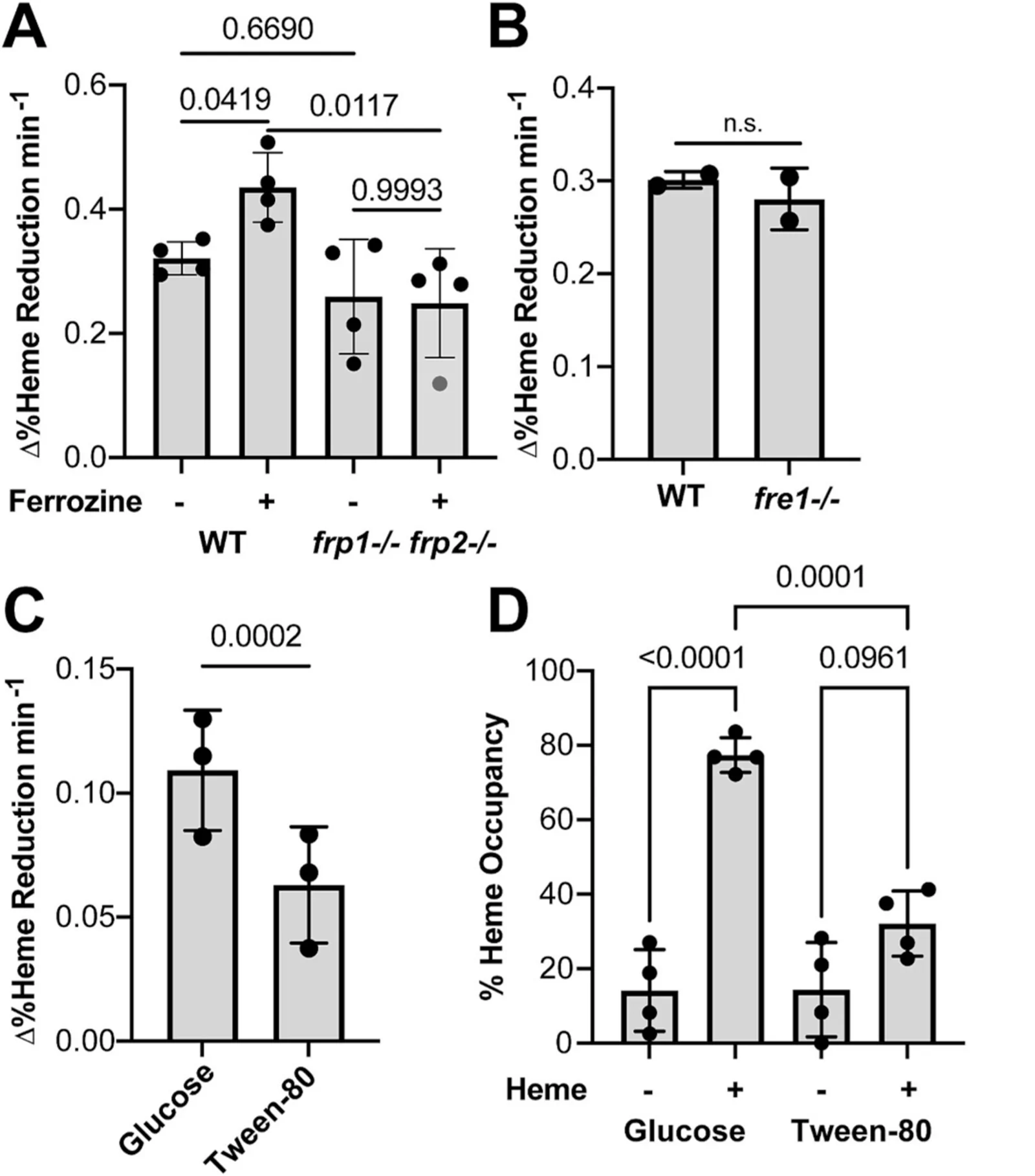
Frp1 and Frp2 as well as carbon source affect cellular heme reductase activity. (A) Effect of ferrozine (1 mM) on the heme reductase activity (Δ% ferrous heme / min) of WT and *frp1*Δ *frp2*Δ mutants. (B) Heme reductase activity of WT and *fre1*Δ cells. (C) Effect of carbon source on heme reductase activity. (D) Effect of carbon source on heme uptake in wild type *C. albicans* expressing the HS1-M7A heme sensor. Cells were cultured in YP media supplemented with 2% glucose or 1% Tween-80, with 1 mM ferrozine, and uptake of hemin (0.5 μM) after 4 h was measured using the heme sensor. In panel A, the data reflect the mean ± standard deviation (SD) of triplicate measurements and the statistical significance assessed by a two-way ANOVA with the Tukey's post-hoc. The data point in red is an outlier that was excluded from the analysis of variance. In panels B and C, the data reflect the mean ± standard deviation (SD) of duplicate or triplicate measurements, respectively, and the statistical significance assessed by a two-tailed t-test. n.s. denotes *p* > .05. In panel D, the data reflect the mean ± standard deviation (SD) of duplicate measurements from duplicate cultures, and the statistical significance was assessed by a two-way ANOVA with the Tukey's post-hoc test. (For interpretation of the references to colour in this figure legend, the reader is referred to the web version of this article.)

Frp1 and Frp2 mediate heme uptake and, based on its similarity to ferric iron reductases, we sought to determine if they contribute to cellular heme reductase activity. Since Frp1 and Frp2 are induced under iron limitation, heme reductase activity was assessed in WT and frp1^−/−^ frp2^−/−^ cells cultured in the absence or presence of 1 mM ferrozine. As shown in Fig. 3A, under iron replete conditions (− ferrozine), when Frp1/2 expression is low, the frp1^−/−^ frp2^−/−^ double knockout showed a modest decrease in heme reductase activity compared to WT cells, albeit the difference was not statistically significant. Under iron limiting conditions (+ ferrozine), when Frp1 and Frp2 are induced (17), there is an elevation in heme reductase activity compared to WT cells under iron replete conditions. Importantly, under iron limiting conditions, there was a statistically significant attenuation in heme reductase activity in frp1^−/−^ frp2^−/−^ cells compared to WT. For comparison, we assessed the heme reductase activity of *C. albicans* lacking a bona fide ferric iron reductase and found that there was no difference in heme reductase activity between WT and fre1^−/−^ cells under iron limiting conditions, which is known to induce FRE1 (10) (Fig. 3B). Thus, taken together, Frp1 and Frp2 in part contribute to cellular heme reductase activity.

Heme reduction is likely dependent on the availability of reducing equivalents like NADH or NADPH. Consequently, energy metabolism and redox homeostasis may play an important role in dictating the extent to which reducing equivalents are available for heme reduction. Carbon source is a major determinant of energy and redox metabolism, which can affect NAD(P)H/NAD(P)^+^ ratios, and *C. albicans* is known to shift metabolism toward fatty acid oxidation during bouts of nutrient limitation (40,41). We therefore determined if there were differences in heme reductase activity if cells were grown on 2% glucose or 1% Tween-80 (source of oleic acid (42,43)), which would force metabolism toward fatty acid oxidation (44,45). As shown in Fig. 3C, cells cultured with 2% glucose exhibited higher heme reductase activity than cells cultured with 1% Tween-80. The diminished heme reductase activity associated with Tween-80 cultured cells correlated with reduced heme uptake as assessed by the heme sensor (Fig. 3D). Together, our results establish that Frp1/2 and carbon source are important determinants of heme reductase activity and uptake. However, the specific metabolic requirements and physiological context for heme reduction need further elucidation.

### 2.4. Saccharomyces cerevisiae exhibits a heme reductase activity that is enhanced when Frp1 is overexpressed

**Fig. 4.**
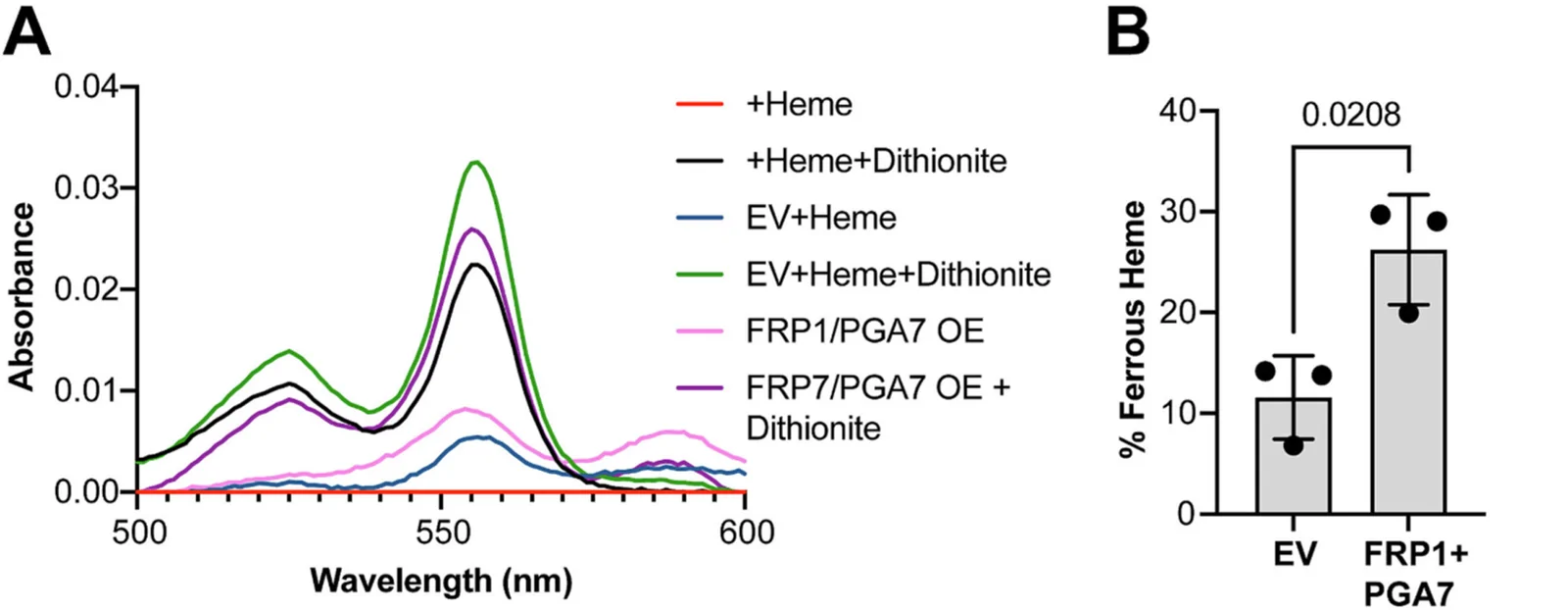
*S. cerevisiae* exhibits a heme reductase activity that is enhanced by overexpression of *C. albicans* Frp1 and Pga7. *S. cerevisiae* strains expressing empty vector (EV) or *FRP1* and *PGA7* have the capacity to reduce extracellular hemin as assessed by the pyridine hemochrome method. (A) 5 μM hemin was incubated with or without 10 mM dithionite and/or with or without 9 OD_600nm_ of the indicated strains for 96 min prior to measurement of the UV/visible spectra with pyridine. (B) % Heme reduced upon exposure to the indicated strains for 96 min. In panel B, the data reflect the mean ± standard deviation (SD) of triplicate measurements, respectively, and the statistical significance assessed by a two-tailed t-test.

We next to sought to determine if other yeast species have the capacity to reduce extracellular heme. *Saccharomyces cerevisiae* (*S. cerevisiae*) which poorly utilizes exogenous hemin as an iron or heme source (46) and lacks the CFEM hemophores of *C. albicans*, was chosen as a counterpoint to *C. albicans* to probe heme reductase activity. As shown in Fig. 4A, a 9.0 OD_600nm_ /mL cell suspension of *S. cerevisiae* has the capacity to reduce hemin. Moreover, overexpression of *C. albicans* heme acquisition factors Frp1 and Pga7 in *S. cerevisiae*, which were previously found to enhance heme uptake of baker's yeast (17), resulted in a 2.5-fold increase in heme reducing capacity (Fig. 4A and B). Together, these results indicate that extracellular heme reduction is a more general feature of yeast and that heterologous expression of *C. albicans* Frp1 in *S. cerevisiae* enhances its heme reducing activity.

## 3. Discussion

Heme is an essential cofactor and nutritional source of iron for the opportunistic fungal pathogen *C. albicans* (16,26,47). *C. albicans* can also utilize heme as a sole source of iron (19). Heme acquisition was previously found to be mediated by CFEM domain containing hemophores that ultimately deliver heme to Frp1 and Frp2, which act as heme receptors that internalize heme through endocytosis (17). Based on the similarity of the Frp proteins to ferric iron reductases (17), we sought to determine if *C. albicans* has a mechanism for reductive heme uptake and if Frp1 and Frp2 were capable of heme iron reduction. Toward this end, utilizing genetically encoded heme sensors, redox inactive metalloporphyrin mimetics of ferric (Ga(III)PPIX) and ferrous (Zn(II)PPIX) heme, and the pyridine hemochrome method to assess heme reduction, we found that: *C. albicans* heme uptake is enhanced when oxygen is limiting or an exogenous reductant is present; *C. albicans* prefers taking up Zn(II)PPIX over Ga(III)PPIX; and *C. albicans* exhibits a heme reductase activity that is in-part dependent on Frp1 and Frp2.

What is the utility of a mechanism for reductive heme uptake? Reductive assimilation of non-heme iron is well established; the insolubility of ferric iron necessitates that it is reduced, leading to more soluble and therefore bioavailable ferrous iron that can be transported (48). However, heme iron, regardless of its oxidation state, is already solubilized by being coordinated to the protoporphyrin IX macrocycle. We propose that heme iron reduction may be important for providing a mechanism for releasing heme from CFEM hemophores. Most available heme for pathogens in mammals is ultimately sourced from hemoglobin, and due to autoxidation, the heme iron oxidation state is mainly +3 (49). CFEM hemophores are selective for ferric over ferrous heme (14). As such, Frp1 and Frp2 may be required to reduce heme so that it can be released from CFEM hemophores prior to internalization. Consistent with this hypothesis, an exogenous reductant like DTT can chemically complement the loss of Frp1 and Frp2. Consequently, Frp1 and Frp2 may play dual roles in heme iron acquisition, both as a heme reductase and as a receptor for heme internalization.

An alternative rationale for heme reduction is that reduced heme is more easily transferred between the factors that mediate its transport and downstream hemoproteins than oxidized heme. Indeed, ligand exchange rates are faster for ferrous over ferric iron (48,50). Our findings that DTT, a cell permeable reducing agent, increases heme sensor occupancy even without addition of exogenous heme is consistent with the possibility that reduced heme is more exchangeable and accessible.

The oxygen dependence of heme uptake may be mediated by multiple mechanisms, in addition to our proposal for a reductive pathway of heme assimilation. For instance, oxygen-dependent heme uptake could be transcriptionally regulated. In the non-pathogenic budding yeast, *S. cerevisiae*, it was previously found that heme transport is induced under heme deficiency or hypoxia, which would lead to heme deficiency due to the oxygen-dependence of heme synthesis (51). In this case, transcriptional induction of proteins that mediate heme import may be responsible for elevated heme uptake, including induction of the putative heme importer PUG1 (51). Moreover, in *C. albicans*, it was previously found that hypoxia induces the transcription of two CFEM domain hemophores, Pga7 and Rbt5 (52), which could also result in elevated heme transport. However, in our experimental system, we found that accumulation of Zn(II)PPIX (Fig. 1B) or Ga(III)PPIX (data not shown) was not affected by oxygen, suggesting that induction of heme transport factors, which often can also transport other metalloporphyrins, is likely not a contributing factor toward oxygen dependent heme uptake.

Reductive heme assimilation pathways may be a broader but underappreciated phenomenon in the microbial world. We previously found that *Mycobacterium tuberculosis* exhibits a heme reductase activity that may be important for heme acquisition and is regulated by the heme synthetic enzyme coproheme decarboxylase (25). In *Staphylococcus aureus* and *Lactococcus lactis*, studies have shown the cell membrane electron carrier menaquinone (MK) could possibly reduce heme directly or indirectly via a still unknown mechanism (33,53). Though, in *L. lactis*, it was not determined if MK-dependent heme reduction was associated with heme uptake. In *S. aureus*, reduction of heme caused induction of reactive oxygen species (ROS) generation, leading to cell membrane damage (33,53). Thus, any benefit of assimilatory heme reduction must be weighed against the detrimental effects of reduced heme in generating ROS. Indeed, we found that cell-reduced heme can be re-oxidized by exposure to oxygen (Fig. 2B), which likely generates ROS like superoxide or hydrogen peroxide. Lastly, we found that the budding yeast, *S. cerevisiae*, also exhibits a heme reductase activity (Fig. 4). Unlike *C. albicans*, *S. cerevisiae* is a poor utilizer of exogenous heme and does not transport heme particularly effectively (46). Nevertheless, the presence of heme reductase activity in baker's yeast offers the promise of being able to conduct rapid high throughput screens to identify putative heme reductases given the genetic tools available, including gene knockout and overexpression libraries. Moreover, overexpression of *C. albicans* heme acquisition factors Frp1 and Pga7, which were previously found to enhance heme uptake in *S. cerevisiae*, (17) resulted in elevated heme reductase activity in baker's yeast (Fig. 4).

Molecular oxygen can re-oxidize heme reduced by *C. albicans* (Fig. 2B), potentially negatively impacting heme uptake through the proposed reductive heme assimilation pathway. Thus, oxygen availability may be a critical determinant of heme uptake. For reference, *p*O_2_ can vary between ~0.5% to ~15% across various tissues (54,55), including ones in which *C. albicans* is known to reside (56,57)such as the skin (~1% - ~5% (54)), liver (~3% - ~5% (54)), gut (~0.4% - 5% (55)) and lung (~13% - ~15% (55)). *C. albicans* can also persist within cells, such as in macrophages (58), where intracellular *p*O_2_ is ~2.5% (59). Exactly how oxygen tension affects heme uptake, especially in physiologically relevant niches, remains to be elucidated.

Electron donors like NADH or NADPH will be important contributors to heme reductase activity. Consequently, energy metabolism and redox homeostasis, which will impact NAD(P)H to NAD(P)^+^ ratios, will likely dictate the magnitude of heme reduction and presumably uptake (60). For instance, when *C. albicans* reside within macrophages or are otherwise nutrient starved, energy metabolism will shift from carbohydrate to fatty acid oxidation, which can in turn lead to changes in NAD(P)H to NAD(P)^+^ ratios (40,41). Indeed, we find that heme reductase activity is dependent on carbon source, i.e. growth on glucose vs. oleic acid, with oleic acid, which promotes fatty acid oxidation, leading to lower heme reductase activity and uptake (Fig. 3C and D). Future work is needed to determine the specific physiological circumstances that might promote reductive heme assimilation.

Altogether, our studies demonstrate that *C. albicans* exhibits heme reductase activity, that this activity may be important for heme uptake, and that Frp1 and Frp2 are in-part required for this activity. Our efforts are now focused on identifying other enzymes that contribute toward heme reductase activity and the physiological context and consequences of reductive heme assimilation, especially as it pertains to virulence.

## 4. Materials and methods

### 4.1. Yeast strains and reagents

*C. albicans* strains used are listed in Table 1. *C. albicans* WT (KC2 = CAF3–1) (61) and isogenic *frp1*Δ *frp2*Δ mutant (KC1410) (17) expressing the heme sensors, WT (SC5314) and isogenic *fre1*Δ strains, and *S. cerevisiae* strain BY4741 expressing *FRP1* and *PGA7* from plasmid KB2566 (17) were previously described. All yeast cells were cultured in YP media (1% yeast extract, 2% bacto-peptone) containing 2% glucose (YPD) or 0.4% to 2% Tween-80 or synthetic complete (SC) media with appropriate drop-out mix. Tween-80 (MPBio) was added from a filter sterilized 10% Tween-80 stock solution in ultrapure water. For growth under iron limiting conditions, the media was supplemented with 1 mM of the iron chelator ferrozine (ThermoScientific). Ferrozine was prepared as a 100 mM stock solution in sterile ultrapure water, diluted into YP or SC media to 1 mM, and heated on a hotplate at 95 °C for 1 h. Cellular iron depletion was confirmed by X-ray fluorescence spectroscopy. Hemin (Sigma-Aldrich) stock solutions were prepared in DMSO (VWR) at concentrations of 10–15 mM and sonicated for 10 min to promote dissolution. Dithiothreitol (DTT) (VWR) stock solutions were prepared as 1 M stock solutions in ultrapure water. Zn^2+^-protoporphyrin and Ga^3+^-protoporphyrin were obtained from Thermo-Scientific and Frontier Specialty Chemicals, respectively. Pyridine and sodium dithionite were obtained from Sigma-Alrich and J.T Baker, respectively.

### 4.2. Labile heme measurements

10 mL cultures of heme sensor expressing cells were grown overnight (14–16 h) in YP media with 2% glucose or 1% Tween-80 to saturation in 50 mL conical tubes, then diluted to an optical density OD_600nm_ of 0.4 and grown for 4 h while shaking at 220 rpm in the indicated YP media containing 2% glucose, 1% Tween-80, and/or 1 mM ferrozine) at 30 °C. Cells were then harvested and diluted to 0.3 OD_600nm_ / mL in 10 mLs of the indicated YP media in a 50 mL loosely capped conical tube and grown for between 1 and 4 h as described in the figure legend with or without 0.5 μM hemin as a standing culture at 30 °C under atmospheric oxygen or a COY anaerobic chamber (95% N_2_ and 5% H_2_). For fluorescence measurements, cells were pelleted, washed twice with ultrapure water, resuspended to OD_600nm_ = 5 OD/mL in PBS, and 200 μLs were placed in replicate in a black 96-well flat-bottom plate. Fluorescence was measured using a Biotek Synergy MX plate reader, with excitation/emission wavelengths of 480/510 nm for eGFP and 580/620 nm for mKATE2. Slit widths for excitation and emission channels were set to 9 nm and 20 nm, respectively. Each measurement was conducted in duplicate for three independent cultures. Background autofluorescence from cells not expressing the heme sensor was subtracted from the eGFP and mKATE2 channels of sensor expressing strains and the eGFP to mKATE2 fluorescence ratios were determined.

Calculation of heme sensor occupancy was done using Eq. (1) (21,63,64).


(1) $$\%Heme\;Occupancy=\frac{\left(R-R_{\min}\right)}{\left(R_{\max}-R_{\min}\right)}*100\%$$

R is the eGFP to mKATE2 fluorescence ratio of the high affinity HS1 or moderate affinity HS1-M7A heme sensors under any given condition, $R_{\text{max}}$ is the heme sensor ratio when the sensor is 100% saturated with heme, and $R_{\text{min}}$ is the heme sensor ratio when the sensor is 0% bound. From in situ heme sensor calibration procedures, it was previously found that HS1 in *C. albicans* and *S. cerevisiae* is quantitively saturated with heme when grown in any iron replete media, e.g. YPD or SC medias (16,21). Thus, $R_{\text{max}}$ is taken from parallel cultures of HS1 expressing yeast in iron replete media. $R_{\text{min}}$ is determined by measuring heme sensor ratios in parallel cultures supplemented with the heme synthetic inhibitor, succinylacetone (250 μM). However, it was previously found that the HS1-M7A, H102A heme sensor, which lacks both heme iron coordinating ligands M7 and H102, has similar eGFP to mKATE2 fluorescence ratios to HS1 or HS1-M7A expressing cells cultured in the presence of succinylacetone. Thus, $R_{\text{min}}$ is taken from parallel cultures of HS1-M7A, H102A expressing yeast.

### 4.3. Total reflection X-ray fluorescence (TXRF)

A Bruker S2 PICOFOX (TXRF) was used to measure metals (64) and ensure that cellular iron was depleted in response to culturing with ferrozine. 3 OD_600_ cell pellets were thawed at room temperature from a −80 °C freezer. Cells were re-suspended in 4 μLs of PBS. For quantification, an internal standard of 1 ppm Ga was spiked into each sample. A 5 ppm Ga stock was prepared from a master stock of 10,000 ppm Ga in 5% HNO_3_ (RICCA CHEMICAL COMPANY). 5 ppm Ga in 5% HNO_3_ was then added to get final concentration 1 ppm in the cell sample. 10 μLs of a silicone solution SERVA (SERVA) was spotted onto the center of a quartz disk and heated on a 120 °C hot plate for 5 min to dry. 4 μLs of the cell suspension was then spotted onto the center of quartz disc and heated again at 120 °C on a hot plate for 5 min to dry. The x-ray fluorescence spectra for each sample was recorded with 1200s measuring time. The identity and concentrations of elements, including iron, were assessed using the S2 Picofox spectral deconvolution software.

### 4.4. Metalloporphyrin uptake assays

Gallium(III)protoporphyrin IX (Ga(III)PPIX) and zinc(II)protoporphyrin IX (Zn(II)PPIX) working stock solutions were made in DMSO at concentrations of 0.5 mM, 1.25 mM, and 2.5 mM. Cells were precultured in YPD media overnight for 14–16 h and the next day were re-diluted to 0.3 OD/mL in 10 mLs. 20 μLs of Zn(II)PPIX or Ga(III)PPIX were added into 10 mLs of YPD cell culture in a 50 mL conical tube to get a final metalloporphyrin concentration of 1.0 μM, 2.5 μM or 5.0 μM. Equal volumes of DMSO were added for samples not treated with metalloporphyrin. Cell cultures were then incubated at 30 °C without shaking for 2 h. Caps were loosened to ensure exchange with air. For experiments comparing metalloporphyrin uptake with and without O_2_, cells were incubated with the indicated metalloporphyrin and concentration at 30 °C without shaking for 2 h in a COY anaerobic chamber (95% N_2_ / 5% H_2_) or in an incubator in air. For experiments comparing metalloporphyrin uptake with and without DTT, cells were incubated with the indicated metalloporphyrin and concentration at 30 °C with or without 1 mM DTT without shaking for 2 h in air. After incubation with the metalloporphyrin, cells were harvested by pelleting at 4000 rpm 4 °C for 7 min. Cell pellets were washed with 900 μLs of pre-chilled PBS + 2% BSA (pH ~ 8.0) twice, with pelleting between wash cycles being accomplished by centrifugation at 14,800 rpm at 25 °C for 1 min. The cells were resuspended in 900 μLs of PBS and optical density at 600 nm (OD_600nm_) was measured to assess cell number.

Fluorescence emission spectra of 200 μLs of a 5 OD_600_/mL suspension was measured between 500 and 700 nm in 1 nm steps using an excitation wavelength of 410 nm (9 nm slitwidth). Ga(III)PPIX and Zn (II)PPIX exhibited λ_max_ for emission at 585 nm and 589 nm, respectively. Autofluorescence of cells treated with the DMSO vehicle only was subtracted from samples that were treated with fluorescent metalloporphyrins and concentration per cell was calculated from Zn(II)PPIX or Ga(III)PPIX standard curves.

### 4.5. Heme reductase activity assays

Heme reductase activity was measured using a modified pyridine hemochrome method (14,37). Briefly, cells were grown overnight (14–16 h) to saturation in 10 mLs of the indicated YP media in a 50 mL conical tube. Cells were diluted to 0.2 OD_600nm_/mL in 50 mLs of the indicated YP media in a 250 mL Erlenmeyer and grown for 6 h to a final density of ~1 OD_600nm_/mL. Cells were harvested by centrifugation at 5000 rpm for 5 min. ~5–10 OD_600nm_ pellets for each sample condition, replicate, and/or mutant was degassed by 3 vacuum and gas cycles in a COY anaerobic chamber airlock and placed in the anaerobic chamber at 30 °C with an atmosphere of 95% N_2_ and 5% H_2_. Cell pellets were washed with 400 μLs of degassed PBS and finally resuspended in 500 μLs of degassed PBS. A 10 mM heme stock solution in DMSO was degassed and diluted into degassed PBS to give a working stock concentration of 500 μM hemin in the COY chamber. Hemin was added to a final concentration of 5 μM to cell samples or degassed PBS as a control and incubated for various times as indicated in the figure legends. After the incubation period, cells were pelleted at maximum speed using a microfuge in the COY chamber and the supernatant was removed. Cells pellets, which had heme visibly associated with it, were then resuspended in 600 μLs of 10% pyridine hemochrome solution (PHS) to extract the cell surface associated heme for measurements of ferrous and total heme. As part of the resuspension process, cells were incubated with 10% PHS for 1–2 min and pelleted by centrifugation in a microcentrifuge at maximum speed, followed by pipetting and removal of the supernatant. 500 μLs 10% PHS supernatant, which solubilized the cell pellet associated heme, was added to a mixture of 125 μLs PBS and 375 μLs 40% PHS in PBS, in a new microcentrifuge tube and mixed. 900 μLs of the mixture was transferred to a capped quartz cuvette in the anaerobic chamber to limit exposure to O_2_. For the non-cell PBS buffer control sample, 450 μLs PBS with 5 μM hemin was aliquoted to a new microcentrifuge tube containing 450 μL 40% PHS (in 0.2 M NaOH). Absorbance spectra from 200 to 600 nm were recorded on a Cary 60 or Olis Clarity UV/vis spectrophotometers. Each sample was measured four times within 15 min to ensure spectral reproducibility and account for any variability due to sample oxidation.

To measure total heme, 10 μLs of 0.5 M sodium dithionite in 0.5 M NaOH was added to samples to fully reduce the heme and obtain the “total heme signal”. All spectra were blank subtracted with 400 μLs of PBS that had incubated in the COY chamber overnight and baseline corrected to account for baseline drift. An extinction coefficient of 34.7 mM^−1^ cm^−1^ at 557 nm was used as a measure of ferrous heme (14). The % ferrous heme signal was obtained by taking the ratio of the signals without and with dithionite.

The PHS was prepared fresh the same day of the heme reductase assay. To make the PHS, 10% or 40% pyridine in 0.2 M NaOH (without potassium ferricyanide) was mixed in a chemical fume hood. PHS was aliquoted into 650 μLs fractions in a microfuge tube, sealed, and placed in the anaerobic chamber. To mitigate the effects of dissolved oxygen, PHS microfuge tube caps were opened periodically over 1–2 h and mixed by pipetting inside the anaerobic chamber prior to use in heme reductase activity measurements.

### 4.6. Statistics

Statistical analyses were performed using GraphPad Prism 10.0 (GraphPad Software, Inc) or KaleidaGraph 5.0 (Synergy Software) and are indicated in the figure legends.

## Figures and Tables

**Table 1:** List of *C. albicans* strains.

Name	Genotype	Reference

KC2 = CAF3–1	*ura3::imm434/ura3::imm434*	(61)
KC643	KC2 <CaURA3>	(61)
KC1170	KC2 < CaURA3 pENO1 CaHS1>	(16)
KC1171	KC2 < CaURA3 pENO1 CaHS1-M7A>	(16)
KC1173	KC2 < CaURA3 pENO1 CaHS1-M7A,H102A>	(16)
KC1410	KC2 < *frp1Δ/frp1Δ frp2Δ/frp2Δ*>	(17)
KC1421	KC1410 <CaURA3>	(17)
KC1418	KC1410 < CaURA3 pENO1 CaHS1>	(17)
KC1420	KC1410 < CaURA3 pENO1 CaHS1-M7A,H102A>	(17)
BWP17	*ura3::imm434/ura3::imm434 iro1::imm434/iro1::imm434 his1::hisG/his1::hisG arg4::hisG/arg4::hisG*	(62)
NKQ4	BWP17 < *fre1Δ/fre1Δ*>	(62)

## Data Availability

All data are included in this publication. If additional information is desired, please contact Amit Reddi at amit.reddi@chemistry.gatech.edu.
